# School-based intervention impacts availability of vegetables and beverages in participants’ homes

**DOI:** 10.3389/fnut.2023.1278125

**Published:** 2023-12-15

**Authors:** Erin A. Hudson, Marissa Burgermaster, Sophia M. Isis, Matthew R. Jeans, Sarvenaz Vandyousefi, Matthew J. Landry, Rebecca Seguin-Fowler, Joya Chandra, Jaimie Davis

**Affiliations:** ^1^The University of Texas at Austin, Austin, TX, United States; ^2^Grossman School of Medicine, New York University, New York, NY, United States; ^3^Stanford Prevention Research Center, Department of Medicine, School of Medicine, Stanford University, Stanford, CA, United States; ^4^Texas A&M AgriLife Research, Temple, NH, United States; ^5^Pediatrics Division, University of Texas MD Anderson Cancer Center, Houston, TX, United States

**Keywords:** school garden, home food environment, home food availability, school-based intervention, nutrition education, Hispanic (demographic), low-income children

## Abstract

As rates of metabolic syndrome rise, children consume too few vegetables and too much added sugar. Because children tend to eat what is available at home, the home environment plays a key role in shaping dietary habits. This secondary analysis evaluated the effects of a school-based gardening, cooking, and nutrition education intervention (TX Sprouts) compared to control on the availability of vegetables, fruit juice, and sugar-sweetened beverages (SSBs) at home. In the TX Sprouts cluster-randomized trial, 16 schools were randomized to TX Sprouts (*n* = 8 schools) or control (*n* = 8 schools) for one academic year. All schools served predominately Hispanic families with low incomes. TX Sprouts built school gardens and taught 18 lessons to all 3rd-5th grade students at intervention schools. TX Sprouts also offered monthly caregiver lessons before and/or after school. Caregivers completed questionnaires pre and post, providing demographics and information about home availability of vegetables, fruit juice, and SSBs. Summary statistics were used to describe the sociodemographic characteristics of participants. Linear regression assessed the change in scores (pre to post) for the food/ beverage availability question. The model was adjusted for the caregiver’s education, employment status, child’s grade, and free or reduced-price lunch eligibility. The analytic sample included 895 participants. Compared to control, the intervention positively changed the home availability of targeted foods and beverages, largely by improving the availability of vegetables and vegetable juice. This study showed that a school gardening, nutrition, and cooking program delivered to elementary children may positively influence the home food environment.

## Introduction

A healthy dietary pattern that includes vegetables and whole fruits and limits added sugar helps maintain health and reduces the risk of metabolic disorders like type 2 diabetes (1–3). Early childhood dietary habits can shape lifelong food preferences and eating behavior (4, 5). In addition, unhealthy eating patterns can lead to metabolic syndrome in childhood (2, 3), increasing the risk of lifelong metabolic disorders (6–8). Yet in the United States, children’s diets fail to meet the recommendations for a healthy dietary pattern, with too few fruits and vegetables and too much added sugar, often in the form of sugar-sweetened beverages (SSBs) (9, 10). As children enter adolescence, they tend to consume even fewer vegetables and more added sugar (11, 12), making early childhood a crucial period to establish healthy dietary habits (4, 5).

Children’s dietary habits are affected by environmental characteristics in their homes and school; therefore, both environments are important intervention points to promote healthy eating habits. At home, children may be influenced by parental modeling (i.e., children choose foods they see caregivers eat) (13). A child’s food choices may also be shaped by their level of nutrition security at home, i.e., whether foods that fit a healthy dietary pattern are regularly available, accessible, and affordable in their homes (14). Nutrition security requires access to and availability of foods and beverages that promote well-being, prevent disease, and align with cultural, social, or dietary preferences (14). Children typically eat what is available in their homes. For example, higher availability of vegetables in the home is associated with higher vegetable consumption in children, and higher availability of SSBs is associated with increased SSB intake (15–17). Although caregivers largely determine the foods and beverages available in the home, a child’s preferences can also influence the foods and beverages their caregiver purchases and brings into the home (15, 16, 18).

School-based gardening interventions are a promising approach to addressing food preferences and promoting healthy dietary patterns in children. Prior studies demonstrate that garden-centered interventions can increase child knowledge and preference for vegetables through exposure, education, and experience growing, harvesting, and tasting vegetables (19, 20) and improve vegetable consumption (6, 21, 22). Many gardening interventions with children also provide classes and resources to caregivers of the participating children, which teach the skills and knowledge to prepare these foods (23). Given their multilayered influence, school-based garden interventions have the potential to improve the home food environment, yet few studies have reported on such findings (24–26).

One such study, Texas! Go! Eat! Grow! (TGEG), was a school-based garden intervention that measured the availability of vegetables, fruit juice, and SSBs in participants’ homes. In the 5-month TGEG pilot, the program improved the availability of vegetables in the homes of third-grade students (24). However, when the TGEG intervention was tested in a cluster randomized controlled trial (cRCT) across 28 schools, TGEG did not measurably change the vegetable, fruit juice, and SSB availability in the participants’ homes (25). This result may be explained by the inconsistent principal and teacher commitment to the program resulting in lower student participation in the cRCT than in the pilot (24, 25).

The TX Sprouts cRCT examined the effect of its school-based gardening, cooking, and nutrition education program on home food environment changes among all 3rd-5th grade children in participating elementary schools (27). TX Sprouts provided a culturally tailored curriculum in elementary schools across central Texas, serving predominantly low-income Hispanic children and their families, and received strong support from teachers and school administrators. TX Sprouts was developed and tested with Hispanic stakeholders, whose feedback was incorporated into the curriculum (28). The nutrition curriculum targets included increasing vegetable consumption, decreasing SSBs and fruit juice, and educating students and caregivers about preparing vegetables in ways aligned with cultural preferences. We previously found that TX Sprouts participants compared to control showed increased vegetable intake (6, 29) and decreased added sugar (6), which aligns with the intervention targets. Based on these nutrition targets and our previous findings, this study aims to determine whether the TX Sprouts intervention compared to control improved the availability of foods and beverages in the home.

## Methods

### Participants and recruitment

This study is a secondary analysis of data from the TX Sprouts program, a cluster-randomized school-based gardening, cooking, and nutrition education intervention. This study enrolled 3^rd^ to 5^th^-grade children and their caregivers from 16 elementary schools in the greater Austin area. Methods and main outcomes for TX Sprouts are published elsewhere (27, 29–31). Briefly, schools were randomized to TX Sprouts intervention (*n* = 8 schools) or control (delayed intervention; *n* = 8 schools) using block randomization for one academic year. In each of the schools recruited, the majority of students were of Hispanic ethnicity (63.6%) and were eligible for the free- and reduced-price lunch (FRL) program (66.0%).

Children and caregivers at the recruited schools were contacted to participate during “Back to School” and “Meet the Teacher” events, via flyers sent home with children, and through teachers’ in-class announcements. All caregiver participants provided written informed consent, and assent was obtained from each participating child. The study was conducted according to the guidelines in the Declaration of Helsinki, and all procedures involving human subjects were approved by the Institutional Review Board of the University of Texas at Austin and the individual school district review boards. The trial is registered at ClinicalTrials.gov (NCT02668744).

### Description of TX Sprouts intervention

The TX Sprouts program was based on the social-ecological-transactional model, which suggests that child behavior and adaptation are shaped by bidirectional influences across levels of ecological systems (32, 33). This model treats the child as nested within micro-systems (e.g., school and family) that reciprocally interact with each other to shape development and behaviors (32, 33). The logic model for the TX Sprouts program proposed that by intervening at the child and school levels, the program could impact the family and home environment (29).

The intervention was implemented in three waves across three academic years from 2016 to 2019. In each wave, the intervention schools each received the same TX Sprouts intervention for one academic year, and the delayed intervention schools received the same intervention the following year. The TX Sprouts research team built a 0.25-acre outdoor teaching garden at each intervention school before the academic year of baseline measurements. At least 6 months before the intervention, Garden Leadership Committees (GLC) were formed and comprised key stakeholders, including teachers and school staff. The GLC frequently met during the planning year to prepare to implement the program long-term. The program provided the schools with all supplies for maintaining the garden. Because the program was culturally tailored to Hispanic populations living in Texas, produce such as squash, peppers, and cilantro were planted and used in the TX Sprouts recipes.

Throughout the academic year, TX Sprouts nutrition and garden educators taught 18-one hour TX Sprouts lessons to each 3rd-5th grade class at the intervention school as part of the students’ typical school day. TX Sprouts provided all material to teach each of these lessons. The 3rd-5th grade teachers attended the lessons, but did not deliver them. The TX Sprouts curriculum was adapted from LA Sprouts (28) and the Junior Master Gardener program developed by Texas A&M AgriLife Extension (34). The student curriculum was designed to be culturally tailored to Hispanic children in Texas, including culturally appropriate recipes, content, and activities.

TX Sprouts lessons aimed to improve a variety of diet-related psychosocial constructs, including increasing nutrition, gardening, and cooking knowledge, self-efficacy, and willingness to try and prefer fruits and vegetables. The curriculum included the following nutrition concepts: (a) cooking/preparing fruits and vegetables consistent with cultural preferences; (b) making nutritious food choices; (c) eating locally produced food; (d) choosing low-sugar beverages made with fresh fruits and vegetables, but not fruit juice, as alternatives to SSBs, like *agua frescas*; and (e) understanding health benefits of fruits and vegetables. Every lesson included either a garden taste test (7 lessons) or a cooking activity (11 lessons), and information and recipe cards were sent home to caregivers following the lessons. The control schools received a delayed intervention in the following academic year, which was identical to the TX Sprouts intervention described above.

During the trial, garden and nutrition educators also taught nine monthly, in-person, 60-min TX Sprouts lessons for caregivers at each school. At the beginning of the year, TX Sprouts educators met with caregivers and school administrators at each school to schedule these classes according to the caregiver preferences at each school. The dates and times varied widely, including mornings, after school, evenings, and weekends based on caregiver preferences. The caregiver lessons were delivered in person in English and Spanish.

The curriculum for the caregivers paralleled the nutrition and gardening topics and activities taught to the children and shared similar skills and knowledge with the caregivers as was previously taught in the student lessons. Each lesson included preparing culturally tailored recipes using fresh produce. These lessons also addressed family shopping, parent modeling, and positive parenting approaches. Incentives to attend the lessons included free meals, produce giveaways, groceries, and free childcare for children and siblings. In addition, children in the TX Sprouts program were invited to attend and encouraged to teach their caregivers how to cook meals with fresh produce, empowering the child to be the champion for healthy changes in the family.

### Instruments and measures

At the beginning and end of the academic school year (pre and post), caregivers were asked to complete a self-administered questionnaire packet, which took 20–30 min to complete. Questionnaires were provided in both English and Spanish and bilingual research assistants were available to assist caregivers in completing them. Caregivers were given a $15 gift card to a local grocery store for completing each questionnaire. The questionnaires asked about the caregiver’s and child’s demographics, eligibility for FRL, caregiver-child grocery shopping behavior, and the availability of vegetables, fruit juice, and SSBs in the home, using the same scale administered in the TGEG trials (24). The questions addressing co-shopping behavior asked whether (yes or no) caregivers did the following activities with their child the previous week: “took your child to the store to get vegetables” or “chose vegetables to buy at the grocery store together.”

The survey concerning home food/beverage availability included seven items that were specifically targeted in the TX Sprouts curriculum: (a) 100% fruit juice; (b) vegetable juice; (c) fresh vegetables; (d) canned, frozen, or dried vegetables; (e) salad; (f) cut up fresh vegetables in a place that is easy for kids to reach; and (g) soft drinks or sugar-sweetened beverages (15). Caregivers were asked to indicate the frequency that each item was available in their home the previous week on a 4-point Likert-type scale: never, some of the time, most of the time, or all of the time. The survey items aligned with the TX Sprouts curriculum, which emphasized eating more vegetables and reducing high-sugar beverage consumption. This same survey about home food/beverage availability has been used in other pediatric populations, reporting a Cronbach’s alpha of 0.7, indicating satisfactory internal consistency (15, 35, 36). A previously published cross-sectional analysis from the TX Sprouts study also incorporated these questions (37). Another Texas-based school garden intervention previously used this survey, which allows clearer comparison of the results.

The responses for each item were converted to numeric values where “never” = 0 and “all of the time” = 3, except for two categories— (1) 100% fruit juice and (2) soft drinks or SSBs—which were coded such that “all of the time” = 0 and “never” = 3. Consistent with the TX Sprouts curriculum, these beverages were reverse-coded due to their deleterious effect on health outcomes when consumed in high amounts (38–42). Scores for each item were summed to yield a composite score for pre and post. A higher composite score indicated a greater availability of vegetables and lower availability of fruit juice and SSBs in the home. The availability scores ranged from 0 to 3 for each item and 0 to 19 for the composite score. A change variable was created representing the difference between pre and post in the composite score and for each of the seven items in the survey.

### Data analysis

Summary statistics were used to describe the sociodemographic characteristics of participants in the intervention and control groups of the analytical sample, using information from the caregivers’ pre-intervention questionnaires. Texas Education Agency data was used to compare the demographics of the eligible students to those in the analytical samples. Chi-square tests and *t*-tests were used to determine differences in demographics between participants in the analytic sample and those excluded due to incomplete survey data. Chi-square tests and t-tests were also used to determine demographic differences between intervention and control. Participant characteristics that significantly differed between intervention and control groups were identified as covariates. Inter-factor correlations were calculated to ensure independence of covariates from each other. Descriptive statistics and *t*-tests were used to analyze the questions regarding caregiver-child co-shopping and evaluate differences pre and post and between intervention and control.

Because this was a cRCT, the necessity of a multilevel model was assessed. A random intercept model in which the intercept varied across schools was fitted and compared to the fixed intercept model. Residual variance at the school level was evaluated, and intraclass correlation coefficients (ICCs) were calculated for the composite score and the score for each of the seven survey items. The intercepts did not vary significantly across the schools, and calculated ICCs further suggested that a multilevel model was not warranted (Supplementary Table 1) (43).

Linear regression was used to assess the change from pre to post in the composite score between intervention and control. The change in each item of the food and beverage availability question was also analyzed with linear regression. The models were adjusted for covariates: caregiver’s education, employment status, child’s grade, and FRL eligibility. Results were considered significant when *p* < 0.05. All analyses were performed using R (version 4.2.0) and R Studio (version 2021.09.0 + 351) software.

## Results

### Fidelity of TX Sprouts intervention

Fidelity to the intervention has previously been described (27, 29). In brief, 100% of the classes were taught to each 3rd-5th grade classroom, less than 1% of the 18 lessons were modified across the eight intervention schools due to school-related interruptions, and 34% of classes were taught indoors due to inclement weather. Caregiver classes were poorly attended; some had to be canceled because no caregivers attended. As a result, only 88.9% of the caregiver classes were taught. Only 7.1% (*n* = 106) of caregivers attended one or more classes, and less than 1% (*n* = 11) attended 50% or more of the nine classes.

### Study sample

**Figure 1:**
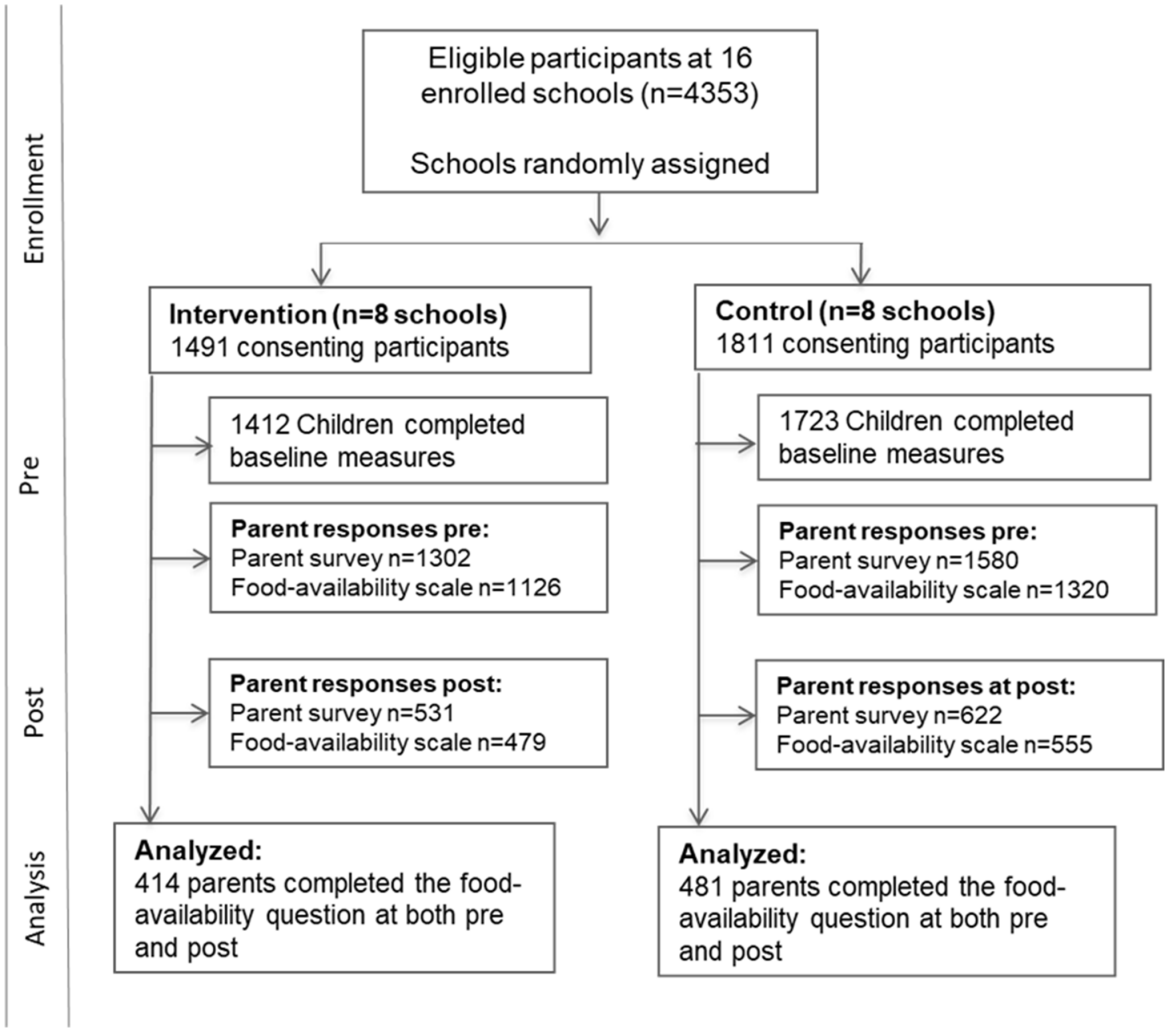
TX Sprouts CONSORT diagram.

Of the 4,353 eligible children at the 16 schools, 3,302 students consented to participate in TX Sprouts. Pre-intervention questionnaire packets were completed by 2,882 caregivers, and 1,153 caregivers completed the packet post-intervention. Given that the 13-page parent questionnaire packet included close to 200 questions and the home availability items included in this analysis were in the middle of the packet, there were many incomplete survey items at both pre- and post-intervention. A complete case analysis, in which inclusion required completion of the 7-item home availability by caregivers at pre- and post-intervention, resulted in an analytical sample of 895 participants (*n* = 414 in intervention; *n* = 481 in control) (Figure 1).

The sociodemographic characteristics of all children eligible to participate (*n* = 4,353), those enrolled in the clinical study (*n* = 3,135), those enrolled in the study with incomplete survey data (*n* = 2,240), and the children in the analytic sample (*n* = 895) are presented in Table 1. There were significantly more children who were female (58%), identified as being Hispanic (62.8%) or Non-Hispanic White (20.2%), and eligible for FRL (66.0%) included in the study than those with incomplete survey data (*p* < 0.001 for all). There was a significant between-group difference in the children’s grade levels between intervention and control, with a higher percentage of older students in the intervention schools because only 4th and 5th grade students were enrolled in some larger schools due to budgetary contraints (Table 1).

**Table 1:** Sociodemographic characteristics of children eligible for TX Sprouts; sociodemographic characteristics of total participating children and caregivers, analytical sample, intervention, and control.

	Eligible participants^a^ (*n* = 4,353)		Enrolled participants (*n* = 3,135)		Participants with incomplete data (*n* = 2,240)		Total analytical sample (*n* = 895)		*p*-value^b^	Intervention (*n* = 414)		Control (*n* = 481)		*p*-value^c^
	No	%	No	%	No	% or ±SD	No	% or ±SD		No	% or SD	No	% or ± SD	
Intervention	1,830	42%	1,412	45.0%	998	44.6%	414	46.3%	0.409	414	100%			
Children’s characteristics														
Sex									<0.001					0.541
Male	2,237	51%	1,485	47.4%	1,111	49.6%	374	41.8%		196	40.7%	178	43.0%	
Female	2,116	49%	1,650	52.6%	1,129	50.4%	521	58.2%		285	59.3%	236	57.0%	
Grade									0.712					0.018*
3rd	1,239	28%	923	29.4%	650	29.0%	273	30.5%		108	56.1%	165	34.3%	
4th	1,554	36%	1,128	36.0%	811	36.2%	317	35.4%		150	36.2%	167	34.7%	
5th	1,560	36%	1,084	34.6%	779	34.8%	305	34.1%		156	37.7%	149	31.0%	
Race or ethnicity^d^									<0.001					0.707
Black or African American	302	7%	264	8.4%	191	8.53%	73	8.16%		36	8.7%	37	7.7%	
Hispanic	2,177	50%	1,869	59.6%	1,307	58.3%	562	62.8%		255	61.6%	307	63.8%	
White (not Hispanic)	498	11%	562	17.9%	381	17.0%	181	20.2%		89	21.5%	92	19.1%	
Eligible for FRL^e^	3,371	77%	1,903	60.7%	1,312	58.6%	591	66.0%	<0.001	276	66.7%	31	65.5%	0.235
Caregivers’ characteristics														
Age			36.89	±6.85	36.8	±6.89	37.1	±6.76	0.181	37.2	±6.55	37.1	±6.95	0.942
Female			2,457	78.4%	1,631	72.8%	826	92.3%	<0.001	389	94.0%	437	90.9%	0.328
Highest level of education									<0.001					0.024*
College degree or higher			552	17.6%	339	15.1%	213	22.8%		91	22.0%	122	25.4%	
Some college			642	20.5%	441	19.7%	201	22.5%		99	23.9%	102	21.2%	
High school diploma or GED			551	17.6%	369	16.5%	182	20.3%		100	24.2%	82	17.0%	
No high school diploma or GED			1,026	32.7%	750	33.5%	276	30.8%		112	27.1%	164	34.1%	
Employment status									<0.001					0.004*
Full-time or more			1,584	50.5%	1,101	49.2%	483	54.0%		235	56.8%	248	51.6%	
Part-time			322	10.3%	213	9.5%	109	12.2%		62	15.0%	47	9.77%	
Retired/not working outside home			814	26%	541	24.2%	273	30.5%		105	25.4%	168	34.9%	
Number of children at home			2.75	±1.21	2.76	±1.22	2.72	±1.18	0.476	2.78	±1.21	2.74	±1.24	0.495

^a^Data gathered from the Texas Education Agency (TEA). ^b^*P*-value for the difference between eligible participants with incomplete survey data and the analytic sample from chi-square tests or independent *t*-tests.

^c^*P*-value for the difference between intervention and control from chi-square tests or independent *t*-tests. ^d^Only three most prominent race and ethnicities were included in this sample to provide a comparison to school-wide data from TEA. ^e^Free- or reduced-price lunches.*Indicates a statistically significant value of *p* < 0.05.

Caregivers were predominantly female (92.3%), and over half worked at least full-time outside the home (54.0%). Caregivers who completed the survey had higher rates of part-time or full-time jobs, had a higher education level, and were comprised of more females compared to those caregivers with incomplete survey data (*p* < 0.001). There were no significant differences between the caregivers’ age or number of children at home in the analytical sample compared to those with incomplete survey data.

Table 1 also compares the sociodemographic characteristics of the caregivers in intervention and control groups. In this study, the intervention had a higher percentage of caregivers working least full-time outside the home than the control (56.8% vs. 51.6%, *p* = 0.004). In addition, the intervention had a lower rate of caregivers with college degrees (22.0% vs. 25.4%) but also a lower percentage of caregivers without at least a high school diploma or GED than the control (27.1% vs. 34.1%) (*p* = 0.024).

At pre-intervention, 70% of caregivers reported that in the previous week they had taken their child to the store to buy vegetables, and 66% of caregivers had chosen vegetables to buy with their child. At post-intervention, the percentage of caregivers reporting that they had co-shopped for vegetables with their child in the past week was similar to pre with 69.9% of caregivers reporting co-shopping with their children. There was no significant difference between intervention and control in either co-shopping question pre or post.

### Home food/beverage availability

**Figure 2:**
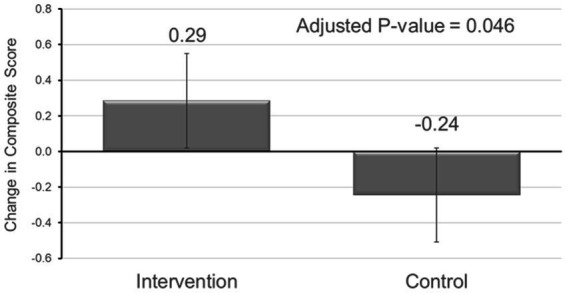
Boxplot showing results of linear regression model examining the effect of TX Sprouts on home availability of vegetables and beverages.

The pre- and post-intervention means with standard deviations are shown in Table 2. The change in the composite score ranged from −11 to 15. The intervention increased home availability of the measured foods/beverages consistent with a healthy dietary pattern compared to control (*β* = 0.528, 95% CI: 0.115, 0.941), as shown in Table 2 and further illustrated in Figure 2. After adjusting for education level and employment status of the caregiver, child’s grade, and eligibility for FRL, the change in the composite score in the intervention remained significant compared to control (*β* = 0.428, 95% CI: 0.009, 0.847).

**Table 2:** Means and standard deviations of responses to home food/beverage availability question, change in score pre and post, and effect of TX Sprouts on that change using linear regression.

Variable	Intervention (*n* = 414)			Control (*n* = 481)				
	Pre Mean ± SD	Post Mean ± SD	Change Mean ± SD	Pre Mean ± SD	Post Mean ± SD	Change Mean ± SD	Change analysis *p*-value	Adjusted^1^ *p*-value
Composite score^2^	11.7 (3.19)	12.0 (3.02)	0.29 (3.07)	12.0 (3.38)	11.8 (3.33)	−0.24 (3.19)	0.012*	0.046*
Individual items								
Fruit juice	1.20 (1.06)	1.36 (1.00)	0.15 (1.05)	1.09 (1.04)	1.33 (1.07)	0.23 (1.04)	0.250	0.434
Vegetable juice	0.80 (0.95)	0.84 (1.00)	0.04 (1.01)	1.01 (1.10)	0.77 (0.98)	−0.23 (1.19)	<0.001*	0.001*
Fresh vegetables	2.27 (0.85)	2.29 (0.84)	0.03 (0.94)	2.36 (0.84)	2.26 (0.87)	−0.10 (0.89)	0.039*	0.122
Canned, frozen, or dried vegetables	2.00 (1.03)	1.98 (1.00)	−0.02 (1.10)	1.95 (1.08)	1.88 (1.08)	−0.07 (1.06)	0.460	0.582
Salad	1.95 (0.97)	1.92 (0.91)	−0.03 (0.98)	2.02 (0.96)	1.94 (0.93)	−0.08 (0.95)	0.442	0.804
Cut up vegetables easy for kids to reach	1.84 (1.07)	1.77 (1.01)	−0.07 (1.16)	1.96 (1.01)	1.81 (1.05)	−0.15 (1.12)	0.295	0.617
Soft drinks or SSBs	1.65 (0.96)	1.84 (0.78)	0.19 (0.91)	1.61 (0.96)	1.77 (0.89)	0.16 (1.00)	0.636	0.480

Adjusted *R*^2^ for adjusted model = 0.016.

*Indicates a statistically significant value of *p* < 0.05. ^1^Adjusted for caregiver’s education, caregiver’s employment status, child’s grade, and child’s eligibility for free- or reduced-priced lunches.

^2^The Composite Score reflects the sum of participant responses to the 7-item food/beverage availability Likert-type question. Consistent with the TX Sprouts curriculum, fruit juice and soft-drinks or SSBs (sugar-sweetened beverages) were coded such that “never” = 3 and “all of the time” = 0; all other items were coded with “never” = 0.

In similar analyses considering each component item, the intervention significantly increased vegetable juice and fresh vegetable availability compared to control (*β* = 0.269, 95% CI: 0.123, 0.415; *β* = 0.126, 95% CI: 0.006, 0.247, respectively). However, after adjustment, the change in vegetable juice availability was the only individual item that significantly improved compared to control (*β* = 0.246, 95% CI: 0.098, 0.394).

## Discussion

This study found that the TX Sprouts school-based gardening, nutrition, and education intervention improved home availability of foods/beverages consistent with a healthy dietary pattern compared to the control group. Specifically, compared to control, the intervention improved the availability of vegetable juice, and there was a trend toward more fresh vegetable availability at home.

As children age, they tend to prefer and consume more sugar-sweetened beverages and fewer vegetables (11, 12). These changes in preferences can influence their household food environment. Though much literature focuses on the role of caregivers in shaping the home food environment, this is not a unidirectional relationship (16, 18). Prior research confirms that children who co-shop with their parents influence their caregivers’ buying behavior at grocery stores (44, 45). This is particularly true with Hispanic children, who co-shop more frequently with their parents than the general U.S. population (44). Similarly, marketing research has found that Hispanic families make more grocery trips per week, and their shopping behavior is more likely to be influenced by their children compared to the general U.S. population (16, 44, 46). In the present study, over two-thirds of all caregivers reported co-shopping with their children within the previous week. The high frequency of caregiver-child co-shopping provides more opportunities for children to influence the food purchased and, therefore, their home food environment (44).

The TX Sprouts program was designed to leverage this interconnectedness between children and the micro-systems to which they belong, such as schools and families using the socio-ecological model (33). The model acknowledges a dynamic, reciprocal influence among children and their micro-systems that influence behavior change (33, 47). Through exposing children to gardening, cooking, and hands-on nutrition education, TX Sprouts compared to control children had increased vegetable intake and decreased SSB consumption, as previously reported (6, 29). This may be explained, in part, by the changes in the child’s home food environment. Similarly, by recognizing the child’s influence on the home food environment, the intervention may have protected against greater adverse changes to the home food environment, such as larger increases in fruit juice availability.

These results may also be attributable to the high fidelity of the TX Sprouts intervention in the participating schools. In one of the few studies reporting the impact of a school-based garden program on the home availability of foods and beverages, the TGEG cRCT trial found no significant changes in the home food and beverage environment using the same survey as TX Sprouts (24, 25). The authors attributed this to low caregiver participation in the home component of the intervention (25). Like TGEG, TX Sprouts had limited caregiver involvement, with less than 7% of participating caregivers attending a single class (29). Though the caregiver involvement was low, the fidelity of the TX Sprouts intervention was high (27, 29). This contrasts with the TGEG cRCT, where student participation varied widely among schools, with only a mean participation rate of 55.7% (25). The high fidelity of the TX Sprouts intervention may explain the program’s success despite the lack of caregiver involvement.

These findings may be further explained by the culturally-tailored recipes and handouts sent home to the intervention households following the student lessons. All schools served predominantly Hispanic families, and TX Sprouts gardens grew culturally-specific produce. In each lesson, students tasted culturally-familiar fresh produce and/or prepared culturally-tailored recipes and were given accompanying handouts and recipes to take home. Past research shows that incorporating culturally familiar foods and recipes can promote acceptance of dietary changes (48, 49). Thus, the resulting improvements in the home environment may have been driven by incorporating culturally tailored TX Sprouts recipes sent home to the caregivers.

The current study has some limitations that must be considered. First, the analytical sample in this complete case analysis (*n* = 895) represents less than one-third of the caregiver participants enrolled in the study, which means data was unavailable for many participants, reducing power and introducing potential bias. There were no significant differences between the caregivers’ age, race/ethnicity, or the number of children or adults in the home in the analytical sample compared to those with incomplete survey data, which mitigates some concerns raised by the attrition rate. However, children in the analytical sample were more likely to be female, Hispanic, and FRL eligible. In addition, the caregivers in the analytical sample had higher education and were more likely to work outside the home compared to those with incomplete survey data. These findings suggest that the analytic sample may have had higher risk factors than eligible participants without complete data. Thus, these variables were included in the adjusted model, minimizing the potential bias due to these differences.

Another weakness of this study is that the food and beverage question was limited to only seven items and did not capture all foods and beverages available in participants’ homes. With over 200 questions in the survey packet, a more detailed examination of all foods available in the home was not possible. It was also not feasible to directly observe foods available in the homes of hundreds of students. Instead, the self-report survey focused on items covered in the curricula and mirrored that used in a similar Texas-based garden intervention, TGEG.

This study was also limited in its duration to one academic school year, and the long-term effects of the intervention on the home food environment were not measured. The improvement in the intervention compared to control was significant but small in this 9-month intervention. A multiple school-year intervention could amplify the at-home impact, particularly if caregivers were more involved. Given the acceptance of remote learning since 2020, online lessons could be a potential avenue to boost caregiver involvement in a longer-term study.

Another limitation was that the sample was predominantly low-income and Hispanic families in Central Texas, so the results may not be generalizable to other populations. However, the study was intentionally designed to target Hispanic children, who are at high risk of metabolic syndrome and similar chronic diseases. As a result, this study sheds light on a potential avenue to improve the home food environment in this population.

In sum, compared to control, the TX Sprouts intervention demonstrated modest improvements in the availability of healthy foods and beverages in the home compared to control over the course of one school year, independent of direct caregiver participation in the program. These findings indicate that children can be agents for positive family nutrition changes. These results further suggest that a school-based program taught only to elementary children may protect nutrition security in families and homes by impacting the availability of foods and beverages that promote well-being, prevent disease, and align with cultural and dietary preferences. Future interventions may build on these findings and incorporate strategies to further empower children to influence the availability of healthier food options in their homes in longer-term education programs, particularly when serving predominantly Hispanic children and their families.

## Data availability statement

The data contains sensitive information concerning minors. De-identified data will be available by request to JD (Jaimie.davis@austin.utexas.edu), and a data sharing agreement will need to be completed. Protocols, analytics, and study material are also available upon request.

## Ethics statement

The studies involving humans were approved by Institutional Review Board at the University of Texas at Austin. The studies were conducted in accordance with the local legislation and institutional requirements. Written informed consent for participation in this study was provided by the participants’ legal guardians/next of kin.

## Author contributions

EH: Conceptualization, Formal analysis, Methodology, Writing – original draft. MB: Conceptualization, Methodology, Supervision, Writing – review & editing. SI: Conceptualization, Formal analysis, Methodology, Writing – review & editing. MJ: Data curation, Investigation, Writing – review & editing. SV: Data curation, Investigation, Writing – review & editing. ML: Data curation, Investigation, Project administration, Writing – review & editing. RS-F: Conceptualization, Writing – review & editing. JC: Writing – review & editing. JD: Conceptualization, Data curation, Funding acquisition, Investigation, Methodology, Project administration, Resources, Supervision, Writing – review & editing.
