# Supplementary material for: School-based intervention impacts availability of vegetables and beverages in participants’ homes
Source: Front Nutr. 2023 Dec 15;10:1278125. doi: 10.3389/fnut.2023.1278125 (PMC10754996; doi:10.3389/fnut.2023.1278125)
Supplement: Supplementary file 1 [file Table_1.docx]

| **Supplementary Table 1.** Intraclass Correlation Coefficients (ICC) and residual variance on school level for composite score and each item (n=16 schools, n=895 caregiver participants) | | |
| --- | --- | --- |
| **Variable** | **ICC^1^** | **Residual Variance on School Level** |
| Composite Score | 0.007 | 0.070 |
| Fruit Juice | 0.017 | 0.019 |
| Vegetable Juice | 0.012 | 0.015 |
| Fresh Vegetables | 0.023 | 0.019 |
| Canned, Frozen, or Dried Vegetables | 0.001 | 0.001 |
| Salad | 0.020 | 0.018 |
| Cut up vegetables easy for kids to reach | 0.005 | 0.007 |
| Soft drinks or sugar-sweetened beverages | 0.031 | 0.029 |
| ^1^ICC calculated on intercept-only model. |  |  |
